# Disseminated Intra-abdominal Actinomycosis Mimicking Colorectal Carcinoma With Diffuse Intra-abdominal Carcinomatosis: A Case Report

**DOI:** 10.7759/cureus.32582

**Published:** 2022-12-16

**Authors:** Najd M AlNowaiser, Abdullah K Almanea, Bushra Al Ahmadi

**Affiliations:** 1 College of Medicine, King Saud Bin Abdulaziz University for Health Sciences, Riyadh, SAU; 2 Department of Pathology and Laboratory Medicine, King Abdulaziz Medical City Riyadh, Riyadh, SAU

**Keywords:** omentum, peritunium, smoking, diabetes, mimic, colorectal carcnoma, disseminated actinomycosis, abscess, actinomyces, pathology

## Abstract

Actinomycosis is a rare opportunistic bacterial infection. It most commonly affects the cervicofacial area and is less common in the gastrointestinal area. Because of the ambiguous clinical pictures, there is a low preoperative diagnosis rate and a high rate of misdiagnoses. In this case report, we have an unusual presentation of disseminated abdominal actinomycosis in a diabetic male patient with no previous history of surgery or trauma. He presented with abdominal pain for two years. Abdominal imaging showed rectal wall thickening with adhesion and attachment to the bladder, and small bowel with omental and peritoneal deposits, mimicking colorectal cancer with abdominal carcinomatosis. Histopathological examination, however, revealed actinomyces infection with no evidence of malignancy. In such cases, the recovery rate with antibiotic therapy is very high in conjunction with surgical resection.

## Introduction

Actinomycete is a non-spore-forming gram-positive rod with branching filaments. These bacteria are a part of normal human flora in multiple systems [1]. Within the gastrointestinal tract, the bacteria can inhabit the appendix, cecum, and colon [1]. Therefore, any disease that can disrupt the normal body barriers in individuals can cause actinomyces infection [2]. Also, immune suppression caused by medications, chemotherapy, HIV, and organ transplantation can contribute to the spread of the infection [3].

The pathogenicity of actinomyces starts with the invasion of normal tissue to the surrounding structures following a mucosal barrier breach, which can trigger an inflammatory process [2]. Usually, the most affected area is the cervicofacial region, which accounts for approximately 50% of infections, followed by the abdominal cavity (20%), and the thoracic area (15% to 20%) [3].

As the bacterial disease progresses, atypical and non-specific symptoms can arise, which can be misdiagnosed as malignancy. In addition, clinical pictures and imaging modalities are not always adequate in differentiating malignancy from inflammatory conditions for a variety of reasons, leading to a 10% lower rate of presurgical diagnosis [1]. In the literature from 2002 to 2022, twenty-eight cases were reported with abdominal actinomyces in patients who underwent surgery, with only two cases that have received a preoperative diagnosis [4]. Another literature review from 1980 to 2018 showed thirteen cases that have been postoperatively diagnosed after histopathological and microbiological assessment [5].

In this case report, we present a case of a 62-year-old man with no prior surgery presenting with disseminated abdominal actinomycosis mimicking colorectal carcinoma with diffuse abdominal carcinomatosis and metastatic disease.

## Case presentation

**Figure 1:**
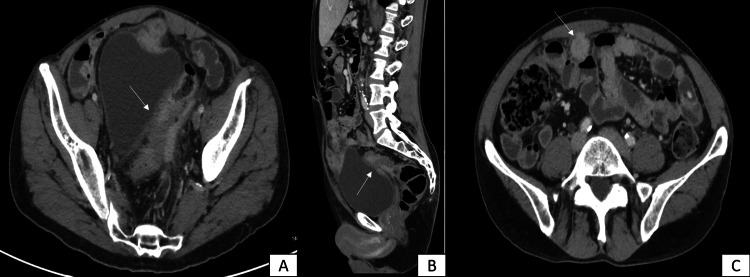
Abdominal CT scan An abdominal CT scan demonstrating the area of the rectal wall asymmetrical thickening with attachment to the bladder dome (A and B) along with the largest peritoneal deposit (C).

A 62-year-old male with a short medical history of diabetes mellitus and long-term smoking (40 pack-years) presented with a history of abdominal pain for the past two years, which has recently gotten worse, and is associated with loss of appetite, and significant weight loss. The patient denied any previous surgical treatment. Physical examination was significant for lower abdominal pain with no tenderness on palpation. All laboratory investigations were within normal limits with no abnormality in the levels of the following tumor markers: CA-125, CA-19.9, and carcinoembryonic antigen. Further investigation was done with computed tomography (CT) scan, which revealed sigmoid demonstrate asymmetric circumferential wall thickening with bladder wall attachment (Figure 1a, 1b). In addition, there were multiple peritoneal and omental deposits in keeping with carcinomatosis, the largest measuring 3 cm in the largest diameter (Figure 1c).

The patient was booked for and underwent an elective exploratory laparotomy with cryo-reductive surgery, omentectomy, rectal, bladder dome, and part of small bowel resection and ileostomy. Surgery revealed an abdominal wall tumor on the right side of the abdomen involving the omentum, urachal, and pelvic disease. There was partial obstruction caused by the tumor.

**Figure 2:**
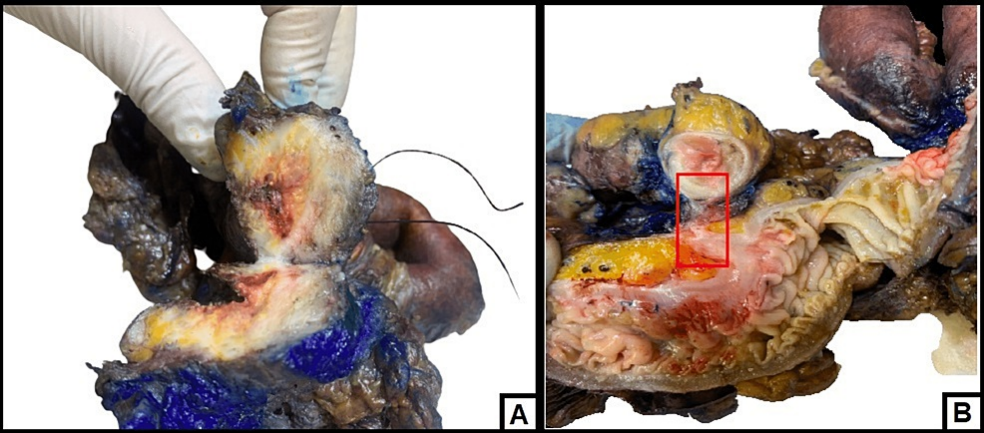
Gross images (A) rectal wall with the lesion extending to the bladder wall "in double suture"; (B) a small adhesion band connecting the rectum to part of the small bowel.

During gross dissection, there were rectal adhesions to both the small bowel (Figure 2a) and bladder wall corresponding to the area of rectal wall thickening (Figure 2b). In addition, there were multiple nodular deposits in the omentum and peritoneum, the largest of which measures 4 cm in largest diameter.

**Figure 3:**
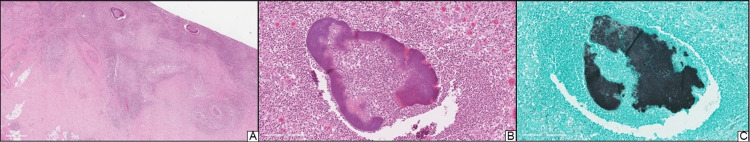
Microscope images Hematoxylin-Eosin (H&E) section (A) shows a low power (x5) of "area of adhesion" between the small and large bowel with fibrosis and inflammation. On higher power (x20) (B), there are mixed acute and chronic inflammatory cells with granulation tissue, abscess formation, and filamentous bacteria with branching rods. These bacteria are staining positively for Grocott’s Methenamine Silver special stain (C).

Under the microscope, the rectal-small bowel adhesion area shows fibrosis, mixed chronic, and inflammatory infiltrate with granulation tissue formation with abscess formation (Figure 3a) and branching filamentous bacterial rods (Figure 3b). The organism stained for Grocott’s Methenamine Silver (GMS) (Figure 3c). There was diffuse intra-abdominal involvement to the bladder wall, mesenteric, omental fat, and peritoneum. The diagnosis of diffuse intra-abdominal actinomycosis was rendered.

As a result of the histopathological diagnosis, the patient received intravenous penicillin treatment for 7 days during his hospitalization, followed by oral penicillin treatment for 30 days at home following his discharge, then will continue a course of 9 months.

## Discussion

In this case, the symptoms were similar to those of actinomycosis, a disease that exhibits a slow progression and nonspecific symptoms such as bowel obstruction [1]. There are several factors that may contribute to the development of abdominal actinomycosis, including abdominal surgery and trauma [2]. Few cases have reported the incidence of abdominal actinomycosis in patients who only had diabetes mellitus and have not undergone any previous surgery [6,7]. Having diabetes mellitus increases a patient’s risk of contracting gastrointestinal infections [8]. According to retrospective studies conducted among 41 patients with abdominal actinomycosis, seven cases showed an association with diabetes [4,5].

The patient has also been smoking one packet a day for the past 40 years. It is known that smoking affects the immune system response, thereby increasing the vulnerability of the mucosa to invasion by the pathogen and the occurrence of infections [9]. Moreover, immune suppression can also contribute to the spread of the infection [3]. There is no literature linking smoking with abdominal actinomyces; however, there is a case reported in 2022 where a smoker developed pulmonary actinomyces that were caused by triggering inflammation [10]. It is necessary to perform further research to ascertain whether the association between smoking and opportunistic infections including actinomycosis can be established.

It is challenging to diagnose actinomycosis. In the absence of typical symptoms, cultures will not be performed to rule out the possibility of bacterial origin. Since actinomycosis can mimic malignancy, inflammation, and infection, it is difficult to distinguish between the two using a CT scan [1]. It is not uncommon for patients to have a low rate of preoperative diagnosis; therefore, an exploratory laparotomy is often the only method to confirm the diagnosis. Biopsy and histopathology are definitive for the diagnosis of digestive tract actinomycosis [2].

## Conclusions

Actinomycosis is rare in the gastrointestinal tract. Radiological images do not provide any specific information. Therefore, misdiagnoses in patients with atypical signs and symptoms are quite common. It is warranted that clinicians should suspect actinomycosis as part of their differential diagnosis, especially in patients with major risk factors. A course of antibiotics is the treatment, and an early diagnosis may significantly reduce the need for surgery and the complications associated with the disease.
